# The effects of short-term calorie restriction on mutations in the spleen cells of infant-irradiated mice

**DOI:** 10.1093/jrr/rrz078

**Published:** 2020-01-07

**Authors:** Saori Kakomi, Takafumi Nakayama, Yi Shang, Chizuru Tsuruoka, Masaaki Sunaoshi, Takamitsu Morioka, Yoshiya Shimada, Shizuko Kakinuma, Akira Tachibana

**Affiliations:** 1 Graduate School of Science and Engineering, Ibaraki University, 2-1-1 Bunkyo, Mito, Ibaraki 310-8512, Japan; 2 National Institute of Radiological Science, National Institutes for Quantum and Radiological Science and Technology, 4-9-1 Anagawa, Inage-ku, Chiba 263-8555, Japan

**Keywords:** calorie restriction, *gpt* delta mouse, infant exposure, mutagenesis, oxidative stress, X-rays

## Abstract

The risk of cancer due to exposure to ionizing radiation is higher in infants than in adults. In a previous study, the effect of adult-onset calorie restriction (CR) on carcinogenesis in mice after early-life exposure to X-rays was examined (Shang, Y, Kakinuma, S, Yamauchi, K, et al. Cancer prevention by adult-onset calorie restriction after infant exposure to ionizing radiation in B6C3F1 male mice. Int J Cancer. 2014; 135: 1038-47). The results showed that the tumor frequency was reduced in the CR group. However, the mechanism of tumor suppression by CR is not yet clear. In this study, we examined the effects of CR on radiation-induced mutations using *gpt* delta mice, which are useful to analyze mutations in various tissues throughout the whole body. Infant male mice (1-week old) were exposed to 3.8 Gy X-rays and fed a control (95 kcal/week/mouse) or CR (65 kcal/week/mouse) diet from adult stage (7-weeks old). Mice were sacrificed at the age of 7 weeks, 8 weeks and 100 days, and organs (spleen, liver, lung, thymus) were harvested. Mutations at the *gpt* gene in the DNA from the spleen were analyzed by using a *gpt* assay protocol that detects primarily point mutations in the *gpt* gene. The results showed that mutation frequencies were decreased in CR groups compared with non-CR groups. Sequence analysis of the *gpt* gene in mutants revealed a reduction in the G:C to T:A transversion in CR groups. Since it is known that 8-oxoguanine could result in this base substitution and that CR has an effect of reducing oxidative stress, these results indicate that the suppression of oxidative stress by CR is the cause of the reduction of this transversion.

## INTRODUCTION

Children are considered to be more highly sensitive to the carcinogenic effects of ionizing radiation than adults. Studies of atomic bomb survivors and many epidemiologic studies of childhood exposure to radiation for treatment of diseases have demonstrated that exposure at a young age results in a higher risk of cancer compared with exposure during adulthood [1, 2]. In addition, because children have a longer life expectancy, the probability of expressing radiation damage is much higher. Since the risk of radiation-related cancer in children appears to continue throughout life, it is crucial to develop strategies for preventing children from radiation-induced cancer.

Cancer is a complex multistage disease associated with the accumulation of multiple gene mutations that cause a deregulation of cell proliferation and differentiation [3]. The multiple steps of carcinogenesis reflect genetic alterations that drive the progressive transformation of normal cells into malignant cells. Calorie restriction (CR) has been shown to decrease spontaneous and chemical carcinogen-induced tumors [4]. CR has also been reported to reduce the incidence of radiation-induced tumors in rats and of radiation-induced leukemia in mice [5–7]. It was also reported that CR reduced the incidence of radiation-induced myeloid leukemia in mice, and that CR, even when started after irradiation, was effective in leukemia reduction [8]. Therefore, CR appears to affect not only the initiation phase but also the promotion phase of carcinogenesis. Although these results indicate that CR is very effective in cancer prevention, CR is not appropriate for children because it might cause malnutrition. To circumvent this difficulty, the effects of adult-onset CR on carcinogenesis in mice irradiated at infant age was examined [9]. Mice were irradiated with X-rays at the age of 1 week (infant) and fed on calorie-controlled diets from the age of 7 weeks (young adult). The results showed that CR reduced the risk of some tumors, e.g. hepatocellular carcinoma, late-occurring non-thymic lymphoma (TL) and lung tumor, indicating that adult-onset CR was effective in suppressing late-occurring tumors caused by childhood exposure. These results suggest that CR might be an effective protocol to suppress the carcinogenic incidence even at later stages after radiation exposure.

A number of studies have shown that a moderate CR might slow ageing, extend life-span and delay the onset of age-associated diseases [4, 10]. CR is also known to cause several metabolic adaptations, such as a decrease in the production of growth factors and anabolic hormones [11, 12], reduction in oxidative stress [13] and reduction in inflammation [14]. In addition, CR affects multiple processes that are involved in the pathogenesis of cancer, for instance, DNA repair processes, apoptosis and autophagy [4]. It has been revealed that there are multiple pathways for nutrient-sensing, e.g. insulin signaling, mammalian target of rapamycin, AMP kinase and sirtuins. These pathways may modulate many cellular signal transduction pathways and transcription factors, which in turn regulate further cellular functions described above [4, 15]. For instance, the forkhead box O transcription factors, which modulate cellular responses including apoptosis, autophagy, cell cycle arrest and stress resistance, are regulated by signaling pathways, such as insulin signaling and sirtuins [16]. However, the molecular basis of the tumor-suppressing effects of CR has not been fully elucidated yet.

Because genetic alterations are involved in many steps during cancer formation and it was indicated that CR affects the promotion stage rather than the initiation stage [17], we assume that adult-onset CR might affect the mechanism of mutagenesis at the progression stage of tumorigenesis after infant irradiation, resulting in the modification of tumor formation. There are several reports that indicated that CR decreased age-dependent accumulation of mutations in rodents by detecting mutations either at the endogenous *Hprt* gene or at the transgenes in mice that harbor a bacterial gene [18–20]. It was also shown that CR reduced mutant fractions induced at the *Hprt* gene by chemical carcinogens in rats [21, 22]. To elucidate the effects of adult-onset CR on radiation-induced mutagenesis, we examined mutations in infant-irradiated mice subjected to CR following the protocol used in the previous tumorigenesis study [9]. For the analysis of mutations in mouse body, we used the *gpt* delta transgenic mouse, which is useful for the detection of both point mutations and deletions [23–25]. In this study, mutations at the *gpt* gene in the spleen from infant-exposed mice were analyzed to clarify the effect of short-term CR on mutations in the irradiated mice, because the *gpt* gene has been shown to be suitable for the detection of a variety of mutations, from base substitutions to deletions [26, 27]. We found that CR decreased mutation frequency at the *gpt* gene, and suggest that the reduction in oxidative stress is responsible for the suppression of mutation frequencies.

## MATERIALS AND METHODS

### Mice

Male B6C3F1 *gpt* delta mice were obtained by mating male C3H/HeNCrlCrj mice (Charles River, Kanagawa, Japan) and female C57BL/6JJmsSlc-Tg (*gpt* delta) mice (Japan SLC, Shizuoka, Japan). Mice were maintained under the conditions of a 12 h dark–light cycle, a temperature of 23 ± 2°C and 50 ± 10% humidity. The mice were weaned at 4 weeks of age and fed a standard diet (MB-1; Funabashi Farm Co., Ltd., Chiba, Japan) until 7 weeks of age.

### Experimental procedure

This study was conducted under the same conditions as the previous experiment [9]. The mice were divided randomly into four groups: (i) non-irradiated with a 95 kcal/week diet (0 Gy-95 kcal group); (ii) non-irradiated with a 65 kcal/week diet (0 Gy-65 kcal group); (iii) 3.8 Gy-irradiated with the 95 kcal/week diet (3.8 Gy-95 kcal group); (iv) 3.8 Gy-irradiated with the 65 kcal/week diet (3.8 Gy-65 kcal group). Irradiation was carried out using a Pantak X-ray generator (Pantak Ltd., East Haven, CT) at 1 week of age and CR started at 7 weeks of age, as previously described [9]. The diets consisted of two different calorie-controlled regimens, 95 and 65 kcal/week/mouse. Details about the food composition and feeding strategy are as described previously [8]. Mice were sacrificed at the age of 7 weeks, 8 weeks and 100 days, and organs (spleen, liver, lung, thymus) were harvested and stored in a frozen state. All experimental procedures were approved by the Animal Experimental Committee (No. 07–1080) and conducted according to the Guidelines for Animal Welfare and Experimentation of the National Institute of Radiological Sciences of Japan.

### *gpt* mutation assay

The *gpt* assay was conducted following the previously published protocol [23, 28, 29]. Briefly, genomic DNA was extracted from the spleen using a RecoverEase DNA isolation kit (Agilent Technologies, Santa Clara, CA, USA). The λEG10 transgene was rescued from the genomic DNA by using a Transpack Packaging Extract (Agilent Technologies). The rescued λEG10 phage was incubated with *Escherichia coli* YG6020 at 37°C for 20 min. After incubation, *E. coli* was incubated at 37°C with vigorous agitation for 30 min. *E. coli* was mixed with 0.6% molten soft agar with or without 25 μg/ml 6-thioguanine (6-TG, Tokyo Chemical Industry Co., Tokyo, Japan) and the entire contents were poured onto M9 plates containing 25 μg/ml chloramphenicol (Cm, Wako Pure Chemical Industries, Osaka, Japan) and 25 μg/ml 6-TG (M9 + Cm + 6-TG plates) or M9 plates containing chloramphenicol only (M9 + Cm plates), respectively, and incubated at 37°C for 3–4 days. The number of colonies appearing on the M9 + Cm plate provided the total number of *E. coli* cells harboring the *gpt*-carrying plasmid, and the number of colonies on the M9 + Cm + 6-TG plates provided the number of *E. coli* cells harboring the mutant *gpt-*carrying plasmid. Colonies on the M9 + Cm + 6-TG plates were subjected to colony PCR for the *gpt* gene.

### Polymerase chain reaction and DNA sequencing analysis of *gpt*

A 739 bp DNA fragment containing the *gpt* gene was amplified by polymerase chain reaction (PCR) from the mutant colony DNA as described previously [29]. PCR products were purified with ExoSAP-IT PCR Product Cleanup Reagent (GE Healthcare Bio-Sciences, Piscataway, NJ, USA). The cycle sequencing reaction was performed using Big Dye Terminator v3.1 (Applied Biosystems, Foster City, CA, USA) with the sequencing primer (5′-ATC TCT ATA ATC TCG CGC AAC C-3′). The analysis was carried out with an ABI PRISM 3100 Genetic Analyzer (Applied Biosystems) according to the manufacturer’s instructions. Oligonucleotide primers were obtained from Hokkaido System Science (Sapporo, Japan).

### Calculation of mutation frequency and clonality

Sequencing analysis revealed that some mutants obtained from the same mice contained the identical sequence alteration, and we considered these mutants containing the same mutation as a clone which was derived from a single mutational event. Mutation frequency was calculated by dividing the number of independent mutations by the number of colonies appearing on M9 + Cm plates.

Clonality was calculated as the ratio of clonal (total-independent) mutations to the total mutations [30, 31].

### Statistical analysis

The statistical significance of mutation frequencies was evaluated with the Mann-Whitney’s U test. *P* < 0.05 was considered statistically significant.

## RESULTS

### Effects of short-term CR diet on body weight and spleen weight

**Fig. 1:**
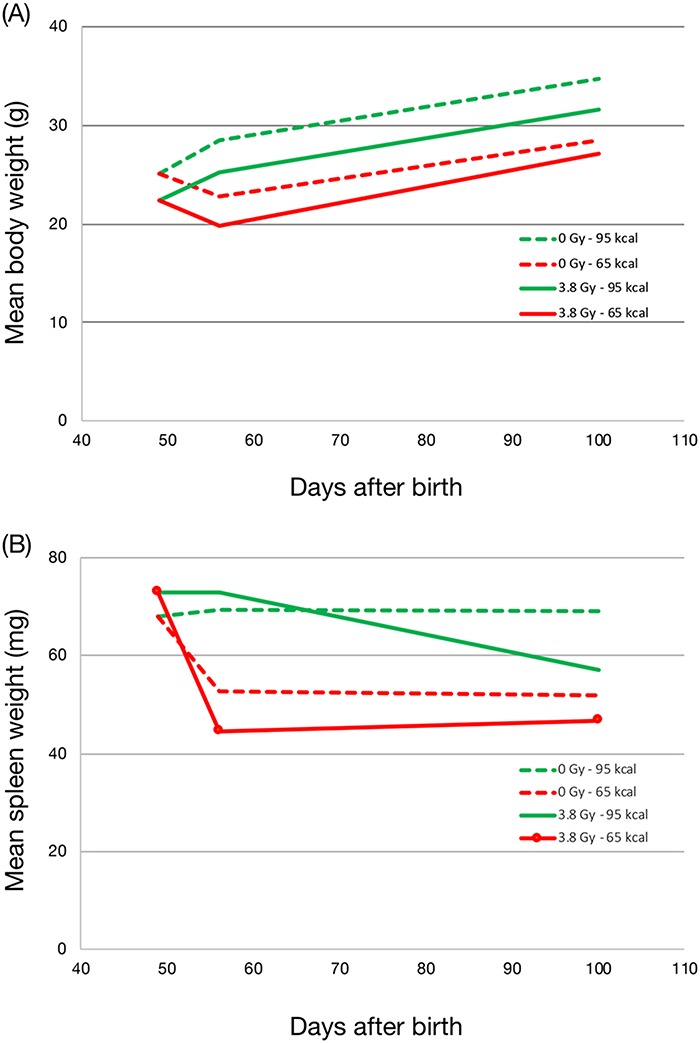
Effect of calorie restriction on body weight (**A**) and spleen weight (**B**). Mice were fed on either 95 kcal/week (green lines) or 65 kcal/week (red lines) diets from the age of 7-weeks and sacrificed at the ages of 7-weeks-old, 8-weeks-old and 100-days-old. Body weights and spleen weights were monitored at the time of sacrifice. Solid line: mice irradiated with 3.8 Gy X-rays; dotted line: unirradiated mice.

The mean body weight for each of the experimental groups was gauged every week to ensure that CR was conducted properly. Fig. 1A shows the mean body weight at the time of sacrifice. As has been reported previously, the growth differences between the 95 and 65 kcal diet groups were apparent even at the age of 8 weeks, i.e. 1 week after starting CR. The mean body weight of CR (65 kcal diet/week) groups was lower than that of the non-CR (95 kcal diet/week) groups with or without irradiation. The spleen weight was also measured at the time of sacrifice. As shown in Fig. 1B, the spleen weight decreased markedly in CR groups 1 week after CR was initiated, irrespective of being irradiated or not. The effect of CR on the spleen weight was more prominent in the irradiated group (3.8 Gy-65 kcal) than in the non-irradiated group (0 Gy-65 kcal). The mean spleen weight remained virtually unchanged until the age of 100 days, except for the irradiated non-CR group (3.8 Gy-95 kcal) in which the mean spleen weight decreased. These results indicate that CR caused decreases in both the body weight and the spleen weight immediately after CR was initiated.

### Effects of calorie restriction on mutation frequency

To investigate the effect of CR on mutations in the spleen of infant-irradiated mice, we analyzed mutations at the *gpt* gene by using the 6-TG selection system. The total number of colonies on non-selective plates and the number of 6-TG-resistant colonies are summarized in Table 1. For further analysis, the *gpt* genes in the 6-TG-resistant mutants obtained were sequenced. We found that some mutants obtained from the same mice contained the identical sequence alteration at the same site in the *gpt* gene. We considered that these mutants with recurrent mutations derived from the tissue of a single animal could be the result of clonal expansion. Based on the number of independent mutants, we calculated the mutation frequency and clonality as shown in Table 1.

**Table 1:** The gpt mutation frequencies in the spleen of X-irradiated or unirradiated mice at the indicated age

Dose	Age	Calories	Animal ID	No. colonies (x10^6^)	No. mutant colonies	No. independent mutations	Mutation frequency (x10^−6^)	Average (x10^−6^)	Clonality (%)	Average (%)
0 Gy	7 w	(A*d libitum*)	S29984–1	0.91	11	8	8.79	5.09	27.3	9.1
			S29984–2	1.16	6	6	5.17		0.0	
			S29984–3	2.28	3	3	1.32		0.0	
	8 w	95 kcal/w	S31779–1	1.45	6	6	4.14	3.51	0.0	0.0
			S31779–2	1.39	4	4	2.88		0.0	
		65 kcal/w	S29985–1	3.14	13	13	4.14	5.07	0.0	0.0
			S29985–3	1.00	6	6	6.00		0.0	
	100 d	95 kcal/w	S32826	0.25	4	4	16.00	13.39	0.0	0.0
			S32827	0.29	1	1	3.45		0.0	
			S32828	0.25	2	2	8.00		0.0	
			S32829	0.21	2	2	9.52		0.0	
			S32830	0.10	3	3	30.00		0.0	
		65 kcal/w	S32832	0.21	2	1	4.76	7.76	50.0	20.8
			S32833	0.33	2	2	6.06		0.0	
			S32834	0.26	3	3	11.54		0.0	
			S32835	0.23	3	2	8.70		33.3	
3.8 Gy	7 w	(*Ad libitum*)	S29980–1	4.08	25	20	4.90	4.82	20.0	16.7
			S29980–2	8.00	10	7	0.88		30.0	
			S29980–3	0.23	2	2	8.70		0.0	
	8 w	95 kcal/w	S29982–1	3.52	24	21	5.97	6.80	12.5	13.2
			S29982–2	1.82	19	17	9.34		10.5	
			S29982–3	0.98	6	5	5.10		16.7	
		65 kcal/w	S29981–1	1.10	4	4	3.64	3.39	0.0	5.8
			S29981–2	11.27	12	11	0.98		8.3	
			S29981–3	1.80	11	10	5.56		9.1	
	100 d	95 kcal/w	S32836	2.63	5	4	1.52	11.73	20.0	4.0
			S32837	0.16	2	2	12.50		0.0	
			S32838	0.14	4	4	28.57		0.0	
			S32839	1.25	3	3	2.40		0.0	
			S32840	0.22	3	3	13.64		0.0	
		65 kcal/w	S32841	2.35	9	6	2.55	6.50	33.3	11.1
			S32842	2.60	5	5	1.92		0.0	
			S32843	2.07	3	3	1.45		0.0	
			S32844	0.25	3	3	12.00		0.0	
			S32845	0.48	9	7	14.58		22.2	

**Fig. 2:**
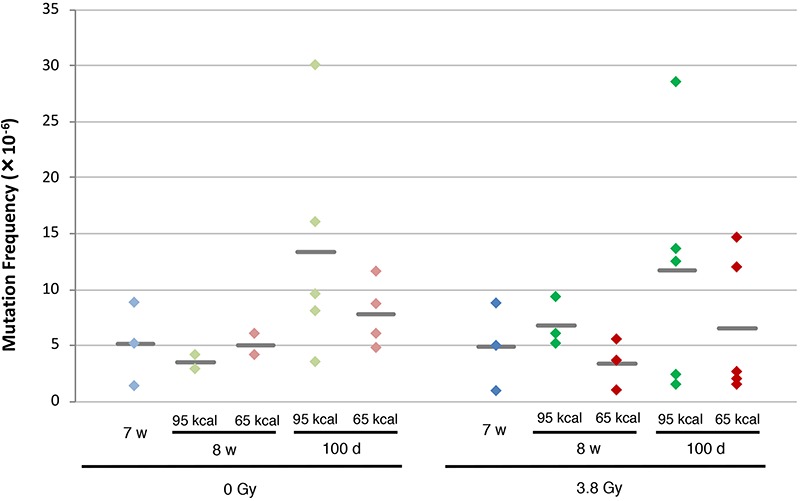
Mutation frequency at the *gpt* gene in the spleen of *gpt* delta mice. The results presented in Table 1 are plotted. Diamonds (green: 95 kcal/week; red: 65 kcal/week) represent the frequency for an individual mouse, and grey bars represent the average frequencies for the groups.

The mutation frequencies in Table 1 are shown in Fig. 2. We did not detect any effect of X-irradiation on the mutation frequency, since there is no significant difference in mutation frequencies between irradiated non-CR groups and corresponding unirradiated non-CR groups. There was an increase in mutation frequencies as the age of mice increased among non-CR groups.

The mutation frequencies of CR groups showed a tendency to be lower than those of the corresponding non-CR groups, although the differences were not statistically significant. The average mutation frequencies of the irradiated non-CR group (3.8 Gy-95 kcal) were 6.80 x 10^−6^ and 11.73 x 10^−6^, at the age of 8 weeks and 100 days, respectively, whereas those of the irradiated CR group (3.8 Gy-65 kcal) were 3.39 x 10^−6^ and 6.50 x 10^−6^, at the age of 8 weeks and 100 days, respectively. Similar tendency was also observed between the unirradiated non-CR group (0 Gy-95 kcal) and unirradiated CR group (0Gy-65 kcal) at the age of 100 days. These results indicate that short-term CR up to 50 days initiated at a young adult age might have a tendency to reduce the mutation frequency in infant-irradiated mice, although it is not statistically significant.

Clonalities in the irradiated groups are slightly higher than those in the unirradiated groups, although the differences were not statistically significant. It is supposed that X-irradiation caused cell death in the spleen, followed by clonal expansion of some of the mutated cells in the process of the recovery of the spleen tissue. It appears that CR had no effect on clonality, as the values of clonality of CR groups were similar to those of non-CR groups, irrespective of whether they were irradiated or not.

### Effect of calorie restriction on mutation spectra

**Fig. 3:**
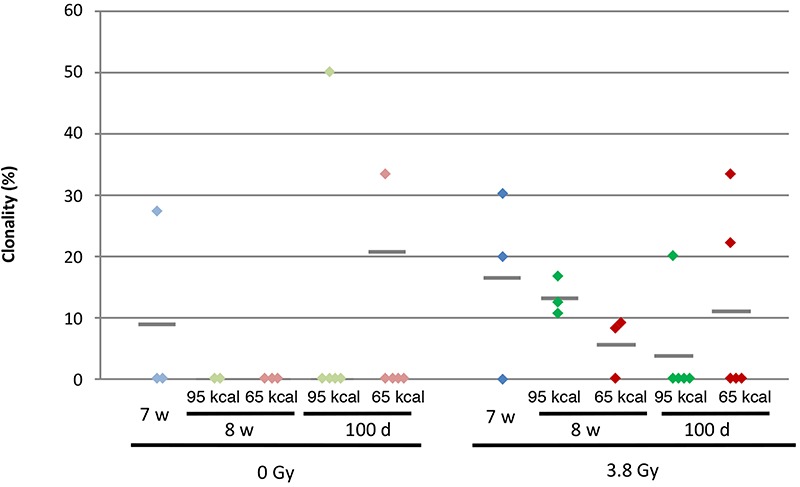
Mutation clonality at the *gpt* gene in the spleen of gpt delta mice. The results presented in Table 1 are plotted. Diamonds (green: 95 kcal/week; red: 65 kcal/week) represent the clonality of an individual mouse, and grey bars represent the average clonalities of the groups.

Types and frequencies of mutations identified at the *gpt* gene are summarized in Table 2 and Fig. 3 (for the sequence alterations in detail, see online supplementary material Tables 1 and 2). The G:C > A:T transition was found predominantly in most cases, as reported in many previous studies [37]. There is not a clear difference in the frequencies of the G:C > A:T mutation between CR groups and non-CR groups, indicating that short-term CR does not have a significant effect on the process of the formation of this mutation. The G:C > T:A transversion is also detected frequently, and the frequencies of this mutation in CR groups showed a tendency to be smaller than those in non-CR groups, especially in irradiated mice. At the age of 8 weeks, the frequency of this mutation in the irradiated non-CR group (3.8 Gy-95 kcal) was 1.27x10^−6^, whereas that in irradiated CR group (3.8 Gy-65 kcal) was 0.41x10^−6^. A similar tendency was also observed at the age of 100 days: 2.20x10^−6^ in 3.8 Gy-95 kcal in comparison with 1.08x10^−6^ in 3.8 Gy-65 kcal. However, these differences were not statistically significant. In unirradiated mice, the frequency of the G:C > T:A mutation was lower in the CR group at the age of 100 days: 2.23x10^−6^ in 0 Gy-95 kcal and 0.97x10^−6^ in 0 Gy-65 kcal. Although the frequencies of other mutations were too low to detect any difference among them, it might be noteworthy that tandem base substitutions were found repeatedly in irradiated mice. Since the mutation frequency of the G:C > T:A transversion showed a tendency to decrease in the CR groups, we concluded that short-term CR started at a young adult age could affect the pathway of radiation-induced mutagenesis in the mouse spleen.

**Table 2:** Spectra of mutations in the *gpt* gene in the spleen of *gpt* delta mice

		7 w (*ad libitum*)			8 w						100 d					
					95 kcal			65 kcal			95 kcal			65 kcal		
Dose	Type of mutation	No. mutations		Frequency^a^ (x10^−6^)	No. mutations		Frequency (x10^−6^)	No. mutations		Frequency (x10^−6^)	No. mutations		Frequency (x10^−6^)	No. mutations		Frequency (x10^−6^)
0 Gy																
	Base substitutions															
	Transitions															
	G:C > A:T	9	(52.9%)	2.69	4	(40.0%)	1.40	4	(21.1%)	1.07	2	(16.7%)	2.23	2	(25.0%)	1.94
	at CpG	3	(17.6%)	0.90	2	(20.0%)	0.70	0	(0.0%)	<0.27	1	(8.3%)	1.12	2	(25.0%)	1.94
	at non CpG	6	(35.3%)	1.80	2	(20.0%)	0.70	4	(21.1%)	1.07	1	(8.3%)	1.12	0	(0.0%)	<0.97
	A:T > G:C	0	(0.0%)	<0.30	0	(0.0%)	<0.35	0	(0.0%)	<0.27	1	(8.3%)	1.12	1	(12.5%)	0.97
	Transversions															
	G:C > C:G	1	(5.9%)	0.30	0	(0.0%)	<0.35	0	(0.0%)	<0.27	0	(0.0%)	<1.12	0	(0.0%)	<0.97
	G:C > T:A	0	(0.0%)	<0.30	2	(20.0%)	0.70	4	(21.1%)	1.07	2	(16.7%)	2.23	1	(12.5%)	0.97
	A:T > T:A	0	(0.0%)	<0.30	0	(0.0%)	<0.35	0	(0.0%)	<0.27	0	(0.0%)	<1.12	0	(0.0%)	<0.97
	A:T > C:G	1	(5.9%)	0.30	1	(10.0%)	0.35	3	(15.8%)	0.80	4	(33.3%)	4.46	0	(0.0%)	<0.97
	Frameshifts															
	1 bp deletion	2	(11.8%)	0.60	0	(0.0%)	<0.35	1	(5.3%)	0.27	3	(25.0%)	3.35	3	(37.5%)	2.91
	1 bp insertion	0	(0.0%)	<0.30	0	(0.0%)	<0.35	1	(5.3%)	0.27	0	(0.0%)	<1.12	0	(0.0%)	<0.97
	2 bp deletion	0	(0.0%)	<0.30	0	(0.0%)	<0.35	0	(0.0%)	<0.27	0	(0.0%)	<1.12	0	(0.0%)	<0.97
	>3 bp deletions	0	(0.0%)	<0.30	2	(20.0%)	0.70	0	(0.0%)	<0.27	0	(0.0%)	<1.12	1	(12.5%)	0.97
	Tandem substitutions	1	(5.9%)	0.30	0	(0.0%)	<0.35	0	(0.0%)	<0.27	0	(0.0%)	<1.12	0	(0.0%)	<0.97
	Complex	3	(17.6%)	0.90	1	(10.0%)	0.35	6	(31.6%)	1.60	0	(0.0%)	<1.12	0	(0.0%)	<0.97
	Total Number of mutants	17			10			19			12			8		
3.8 Gy																
	Base substitutions															
	Transitions															
	G:C > A:T	9	(31.0%)	1.50	12	(27.9%)	1.90	7	(28.0%)	0.95	4	(25.0%)	2.93	8	(33.3%)	2.17
	at CpG	5	(17.2%)	0.83	2	(4.7%)	0.32	5	(20.0%)	0.68	1	(6.3%)	0.73	4	(16.7%)	1.08
	at non CpG	4	(13.8%)	0.66	10	(23.3%)	1.58	2	(8.0%)	0.27	3	(18.8%)	2.20	4	(16.7%)	1.08
	A:T > G:C	1	(3.4%)	0.17	4	(9.3%)	0.63	4	(16.0%)	0.54	0	(0.0%)	<0.73	1	(4.2%)	0.27
	Transversions															
	G:C > C:G	2	(6.9%)	0.33	1	(2.3%)	0.16	1	(4.0%)	0.14	1	(6.3%)	0.73	0	(0.0%)	<0.27
	G:C > T:A	8	(27.6%)	1.33	8	(18.6%)	1.27	3	(12.0%)	0.41	3	(18.8%)	2.20	4	(16.7%)	1.08
	A:T > T:A	0	(0.0%)	<0.17	1	(2.3%)	0.16	0	(0.0%)	<0.14	2	(12.5%)	1.47	3	(12.5%)	0.81
	A:T > C:G	0	(0.0%)	<0.17	2	(4.7%)	0.32	0	(0.0%)	<0.14	1	(6.3%)	0.73	1	(4.2%)	0.27
	Frameshifts															
	1 bp deletion	1	(3.4%)	0.17	5	(11.6%)	0.79	4	(16.0%)	0.54	2	(12.5%)	1.47	2	(8.3%)	0.54
	1 bp insertion	0	(0.0%)	<0.17	0	(0.0%)	<0.16	2	(8.0%)	0.27	0	(0.0%)	<0.73	0	(0.0%)	<0.27
	2 bp deletion	2	(6.9%)	0.33	0	(0.0%)	<0.16	0	(0.0%)	<0.14	0	(0.0%)	<0.73	0	(0.0%)	<0.27
	>3 bp deletions	1	(3.4%)	0.17	4	(9.3%)	0.63	0	(0.0%)	<0.14	0	(0.0%)	<0.73	1	(4.2%)	0.27
	Tandem substitutions	0	(0.0%)	<0.17	3	(7.0%)	0.47	1	(4.0%)	0.14	0	(0.0%)	<0.73	0	(0.0%)	<0.27
	Complex	5	(17.2%)	0.83	3	(7.0%)	0.47	3	(12.0%)	0.41	3	(18.8%)	2.20	4	(16.7%)	1.08
	Total Number of mutants	29			43			25			16			24		

^a^Frequency of each type of mutation was calculated by multiplying the average mutation frequency of the group by the ratio of that mutation.

**Fig. 4:**
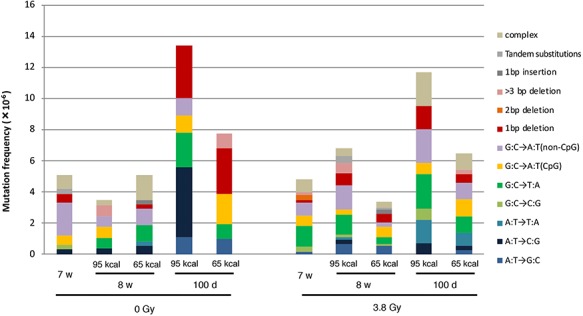
Frequencies of each kind of mutations identified in the *gpt* gene in the spleen of *gpt* delta mice. The data on frequencies of each type of mutation in Table 2 are presented.

## DISCUSSION

We examined the effect of adult-onset CR on the mechanisms of mutagenesis in infant-irradiated mice. The results of the body weight and the spleen weight (Fig. 1) indicated that CR had some effect on the mouse body even at 1 week after CR had started, similar to the previous reports [8, 9]. These results suggest that CR is effective in modifying cellular functions within a short period.

Our analysis of mutations in the *gpt* gene revealed that the mutation frequencies in the irradiated CR groups (3.8 Gy-65 kcal) were lower than those in the irradiated non-CR groups (3.8 Gy-95 kcal) at the age of 8 weeks and 100 days. Because CR started 6 weeks after the mice had been irradiated with X-rays, almost all DNA damage initially caused by X-irradiation should have disappeared when CR was initiated in this study. Therefore, the mutations observed at the age of 8 weeks and 100 days are not likely to be the result of DNA lesions induced by X-irradiation directly, but are presumably caused by DNA lesions generated indirectly after initial damage was repaired. Since cancer is a multistage disease and genetic alterations are observed not only at the early stages but also at the later stages [3], these mutations caused at the later stage might be involved in the progression stage of carcinogenesis. This suggests that CR affected the mutagenesis process at the progression stage of carcinogenesis, which is in agreement with the conclusion of previous studies that suggested that CR affects the promotion phase of tumorigenesis [8, 17].

The analysis of mutation spectra indicated that CR reduced the frequency of the G:C > T:A transversions. It is known that this base substitution results from the mis-pairing of 8-oxoG, a base damage caused by reactive oxygen species, with adenine during the DNA replication process [32]. Therefore, we assumed that the decrease in the production of 8-oxoG resulted in a reduction in the frequency of the G:C > T:A mutation. As it is known that CR reduces oxidative stress in cells and tissues [4, 15, 33], we assume that CR decreased the level of 8-oxoG in mouse body more than 6 weeks after X-irradiation, leading to the reduction in the G:C > T:A frequency.

It is suggested that ionizing radiation causes chronic oxidative stress as well as initial DNA damage, and that this chronic oxidative state might lead to late cellular injury [34, 35]. We suppose that the chronic oxidative stress is responsible for 8-oxoG generation at the later stage, and that CR reduced the chronic oxidative stress resulting in a decrease in the frequency of the G:C > T:A transversion. Thus, CR might affect mutagenesis at the late stage, which may, in turn, decrease the formation of tumors. The chronic oxidative state is also suggested to be related to continuous inflammation, which may also influence the carcinogenic process. Thus, the combined effects of CR on both the gene mutation and the inflammation via the reduction of chronic oxidative stress might contribute to the suppression of tumor formation.

He *et al.* reported that the spectra of age-dependent mutations were not significantly different between the CR group and the non-CR group, concluding that CR did not alter any specific type of mutations but rather affected many types of DNA repair mechanisms and/or DNA damage-prevention systems [20]. Since their study analyzed spontaneous mutations, we assume that the level of oxidative stress was relatively small, and that the effect of CR on oxidative stress might be too small to be identified. On the other hand, as the level of oxidative stress is supposed to be much higher in X-irradiated mice, we could detect the effect of CR on oxidative stress in this study.

In this study, we analyzed the effects of short-term CR on mutations at the *gpt* gene in the spleen of infant-irradiated transgenic mice. Since CR is considered to affect the later stage of carcinogenesis as discussed above, it is of interest to examine the effects of long-term CR on mutagenesis. In addition, it is necessary to analyze the effects of CR in other tissues, as it was reported that there were certain differences among tissues in the effects of CR on the suppression of tumorigenesis [9]. Although we analyzed mutations at the *gpt* gene which detects small mutations such as base substitutions and frameshifts in this study, it is known that large deletions are frequently induced by ionizing radiation [36]. As the *gpt* delta mouse used in this study is suitable for analyzing large deletions [24], we are currently analyzing deletions at the *red*/*gam* genes by using Spi^−^ selection as well as the analysis of gpt mutations.

In conclusion, we found that adult-onset CR decreased the frequency of gene mutations in infant-irradiated mice by reducing oxidative stress. Further investigation would reveal the mechanism of the effect of CR on cellular functions and lead to the development of an effective way to prevent tumor formation.

## Supplementary Material

SupplTable1a1_rrz078

SupplTable2a1_rrz078
