# Supplementary material for: The effects of short-term calorie restriction on mutations in the spleen cells of infant-irradiated mice
Source: J Radiat Res. 2020 Jan 7;61(2):187–96. doi: 10.1093/jrr/rrz078 (PMC7246060; doi:10.1093/jrr/rrz078)
Supplement: SupplTable1a1_rrz078 [file suppltable1a1_rrz078.docx]

Supplementary Table 1. Mutations at the *gpt* gene in the spleen of unirradiated mice

| Age | Calories | Animal ID | Position^a^ | Mutation | No. of mutants | Sequence Alteration^b^ |
| --- | --- | --- | --- | --- | --- | --- |
| 7 W | (Ad lib.) | S29984-1 | 64 | C>T | 2 | AAGCCGACT > AAGCTGACT |
|  |  |  | 92 | G>A | 1 | AAAGGCATT > AAAGACATT |
|  |  |  | 110 | G>A | 3 | AGCCGTGGC > AGCCATGGC |
|  |  |  | 185 | G>A | 1 | TCCAGCTAC > TCCAACTAC |
|  |  |  | 275 | A>C | 1 | GTGGATACC > GTGGCTACC |
|  |  |  | 406 | G>A | 1 | GATTGAACA > GATTAAACA |
|  |  |  | 416-418 | delG | 1 | CCGTGGGATAT > CCGTGGATAT |
|  |  |  | 418 | G>A | 1 | GTGGGATAT > GTGGAATAT |
|  |  | S29984-2 | 8-12 | delA | 1 | AGCGAAAAATACA > AGCGAAAATACA |
|  |  |  | 112 | G>C | 1 | CCGTGGCGG > CCGTCGCGG |
|  |  |  | 201-202 | CC>TT | 1 | ACAACCAGCG > ACAATTAGCG |
|  |  |  | 274 | G>A | 1 | GGTGGATAC > GGTGAATAC |
|  |  |  | 402 | G>A | 1 | CCTGGATTG > CCTGAATTG |
|  |  |  | complex 1 | | 1 | 33-34 delGT; 383-384 AT > G |
|  |  | S29984-3 | 115 | G>A | 1 | TGGCGGTCT > TGGCAGTCT |
|  |  |  | complex 2 | | 1 | 1-14 del(ATGAGCGAAAAATA); 20-21 delTC; 24-25 insT; 29-42 CATGTTGCAGATC > A; 78-79 delTG; 245-247 delAAG; 278-281 CCGG > TA; 435 C > G |
|  |  |  | complex 3 | | 1 | 1-7 ATGAGCG > GT; 33-38 GTTGCA > T; 44 delA; 185 G > T; 253 delA |
| 8 W | 95 kcal | S31779-1 | 106 | A>C | 1 | CGTAAGCCG > CGTACGCCG |
|  |  |  | 110 | G>A | 1 | AGCCGTGGC > AGCCATGGC |
|  |  |  | 184-187 | del | 1 | TTTCC**a**gct**A**CGATC |
|  |  |  | 402 | G>A | 1 | CCTGGATTG > CCTGAATTG |
|  |  |  | 406 | G>A | 1 | GATTGAACA > GATTAAACA |
|  |  |  | 413-428 | del | 1 | ACAGC**cgt**gggatatgggcgt**CGT**ATTCG |
|  |  | S31779-2 | 64 | C>T | 1 | AAGCCGACT > AAGCTGACT |
|  |  |  | 108 | C>A | 1 | TAAGCCGTG > TAAGACGTG |
|  |  |  | 186 | C>A | 1 | CCAGCTACG > CCAGATACG |
|  |  |  | complex 4 | | 1 | 21-22 insA; 25 delT; 28-29 insA |
|  | 65 kcal | S29985-1 | 105-106 | insT | 1 | CGTA\|AGCC > CGTATAGCC |
|  |  |  | 108 | C>A | 1 | TAAGCCGTG > TAAGACGTG |
|  |  |  | 116 | G>A | 1 | GGCGGTCTG > GGCGATCTG |
|  |  |  | 176 | G>A | 1 | GTTTGTATT > GTTTATATT |
|  |  |  | 304 | G>T | 1 | TCGTGAAAT > TCGTTAAAT |
|  |  |  | 345 | A>C | 1 | CAAAACCGG > CAAACCCGG |
|  |  |  | 406 | G>A | 1 | GATTGAACA > GATTAAACA |
|  |  |  | 459 | A>C | 1 | GCTAA- > GCTAC- |
|  |  |  | complex 5 | | 1 | 1 delA; 7 G > T; 20 delT |
|  |  |  | complex 6 | | 1 | 1-2 insG; 34 delT |
|  |  |  | complex 7 | | 1 | 105-106 insT; 232 G > A |
|  |  |  | complex 8 | | 1 | 110 G > T; 414 G > C |
|  |  |  | complex 9 | | 1 | 279 delC; 282-285 TGGT > CG; 288-289 insGT; 291-292 insC |
|  |  | S29985-3 | 15 | C>A | 1 | AATACATCG > AATAAATCG |
|  |  |  | 176 | G>A | 1 | GTTTGTATT > GTTTATATT |
|  |  |  | 185 | G>T | 1 | TCCAGCTAC > TCCATCTAC |
|  |  |  | 423-425 | delG | 1 | ATATGGGCGTC > ATATGGCGTC |
|  |  |  | 437 | T>G | 1 | TTCGTCCCG>TTCGGCCCG |
|  |  |  | complex 10 | | 1 | 110 G > A; 457 T > G |
| 100 d | 95 kcal | S32826 | 150-152 | delG | 1 | AACTGGGTATT > AACTGGTATT |
|  |  |  | 261 | T>G | 1 | TTATTGATG > TTATGGATG |
|  |  |  | 402 | G>A | 1 | CCTGGATTG > CCTGAATTG |
|  |  |  | 406 | G>T | 1 | GATTGAACA > GATTTAACA |
|  |  | S32827 | 345 | A>C | 1 | CAAAACCGG > CAAACCCGG |
|  |  | S32828 | 150-152 | delG | 1 | AACTGGGTATT > AACTGGTATT |
|  |  |  | 310 | T>G | 1 | AATGTATCC > AATGGATCC |
|  |  | S32829 | 86-87 | delG | 1 | CAATGGAAAG > CAATGAAAG |
|  |  |  | 453 | T>C | 1 | CCGGTCGCT > CCGGCCGCT |
|  |  | S32830 | 101 | C>A | 1 | ATTGCCGTA > ATTGACGTA |
|  |  |  | 110 | G>A | 1 | AGCCGTGGC > AGCCATGGC |
|  |  |  | 312 | T>G | 1 | TGTATCCAA > TGTAGCCAA |
|  | 65 kcal | S32832 | 110 | G>A | 2 | AGCCGTGGC > AGCCATGGC |
|  |  | S32833 | 110 | G>A | 1 | AGCCGTGGC > AGCCATGGC |
|  |  |  | 442-443 | delC | 1 | CCCGCCAATC > CCCGCAATC |
|  |  | S32834 | 318-327 | del | 1 | CAAAA**g**cgcacttt**G**TCACC |
|  |  |  | 416-418 | delG | 1 | CCGTGGGATAT > CCGTGGATAT |
|  |  |  | 423-425 | delG | 1 | ATATGGGCGTC > ATATGGCGTC |
|  |  | S32835 | 181 | T>C | 1 | TATTTCCAG > TATTCCCAG |
|  |  |  | 402 | G>T | 2 | CCTGGATTG > CCTGTATTG |

^a^ Positions in the coding sequence of the *gpt* gene are numbered, the A of the first codon being the first nucleotide. In the case of deletions in repeat sequences, the positions of repeats are indicated. For deletions larger than three bases, deleted regions are indicated.

^b^ Alterations from the wild-type sequences to the mutant sequences are indicated for small mutations. Altered bases are underlined in both wild-type and mutant sequences. For deletions larger than three bases, deleted bases are shown in lower case letters. Microhomologies are indicated in bold letters.
