# Supplementary material for: The effects of short-term calorie restriction on mutations in the spleen cells of infant-irradiated mice
Source: J Radiat Res. 2020 Jan 7;61(2):187–96. doi: 10.1093/jrr/rrz078 (PMC7246060; doi:10.1093/jrr/rrz078)
Supplement: SupplTable2a1_rrz078 [file suppltable2a1_rrz078.docx]

Supplementary Table 2. Mutations at the *gpt* gene in the spleen of X-irradiated (3.8 Gy) mice

| Age | Calories | Animal ID | Position^a^ | Mutation | No. of mutants | Sequence Alteration^b^ |
| --- | --- | --- | --- | --- | --- | --- |
| 7 W | (Ad lib.) | S29980-1 | 3 | G>A | 3 | ATGAGCG > ATAAGCG |
|  |  |  | 6 | C>G | 1 | TGAGCGAAA > TGAGGGAAA |
|  |  |  | 7 | G>T | 1 | GAGCGAAAA > GAGCTAAAA |
|  |  |  | 8-12 | delA | 1 | AGCGAAAAATACA > AGCGAAAATACA |
|  |  |  | 27 | G>A | 1 | CCTGGGACA > CCTGAGACA |
|  |  |  | 54-55 | delAC | 1 | GTAAACTCGC > GTAATCGC |
|  |  |  | 64 | C>T | 1 | AAGCCGACT > AAGCTGACT |
|  |  |  | 66-67 | delAC | 1 | GCCGACTGAT > GCCGTGAT |
|  |  |  | 115 | G>A | 1 | TGGCGGTCT > TGGCAGTCT |
|  |  |  | 116 | G>T | 1 | GGCGGTCTG > GGCGTTCTG |
|  |  |  | 139 | G>C | 1 | ACTGGCGCG > ACTGCCGCG |
|  |  |  | 168-176 | del | 3 | GTCGA**ta**ccgtttg**TA**TTTCC |
|  |  |  | 190 | G>A | 1 | CTACGATCA > CTACAATCA |
|  |  |  | 263 | A>G | 1 | ATTGATGAC > ATTGGTGAC |
|  |  |  | 274 | G>T | 1 | GGTGGATAC > GGTGTATAC |
|  |  |  | 401 | G>A | 1 | ACCTGGATT > ACCTAGATT |
|  |  |  | 406 | G>A | 2 | GATTGAACA > GATTAAACA |
|  |  |  | complex 11 | | 1 | 6-7 GG > AT; 52 A > G |
|  |  |  | complex 12 | | 1 | 115 G > A; 455 G > C |
|  |  |  | complex 13 | | 1 | 199-200 delAA, insC |
|  |  | S29980-2 | 7 | G>T | 4 | GAGCGAAAA > GAGCTAAAA |
|  |  |  | 26 | G>T | 1 | ACCTGGGAC > ACCTTGGAC |
|  |  |  | 64 | C>T | 1 | AAGCCGACT > AAGCTGACT |
|  |  |  | 110 | G>A | 1 | AGCCGTGGC > AGCCATGGC |
|  |  |  | 401 | G>T | 1 | ACCTGGATT > ACCTTGATT |
|  |  |  | complex 14 | | 1 | 7 G > T; 20 T > C |
|  |  |  | complex 15 | | 1 | 7 G > T; 401 G > A |
|  |  | S29980-3 | 176 | G>T | 1 | GTTTGTATT > GTTTTTATT |
|  |  |  | 406 | G>T | 1 | GATTGAACA > GATTTAACA |
| 8 W | 95 kcal | S29982-1 | 3 | G>A | 1 | ATGAGCG > ATAAGCG |
|  |  |  | 25 | T>C | 1 | CACCTGGGA > CACCCGGGA |
|  |  |  | 27 | G>A | 4 | CCTGGGACA > CCTGAGACA |
|  |  |  | 82 | C>T | 1 | TGAACAATG > TGAATAATG |
|  |  |  | 86-87 | GG>AA | 1 | CAATGGAAAG > CAATAAAAAG |
|  |  |  | 91-103 | del | 1 | GGAAAggcattattgccgTAAGC |
|  |  |  | 110 | G>A | 1 | AGCCGTGGC > AGCCATGGC |
|  |  |  | 112 | G>T | 1 | CCGTGGCGG > CCGTTGCGG |
|  |  |  | 115 | G>A | 1 | TGGCGGTCT > TGGCAGTCT |
|  |  |  | 116 | G>T | 1 | GGCGGTCTG > GGCGTTCTG |
|  |  |  | 187 | T>A | 1 | CAGCTACGA > CAGCAACGA |
|  |  |  | 244 | G>T | 1 | TGGCGAAGG > TGGCTAAGG |
|  |  |  | 263 | A>C | 1 | ATTGATGAC > ATTGCTGAC |
|  |  |  | 274 | G>A | 1 | GGTGGATAC > GGTGAATAC |
|  |  |  | 296-329 | del | 1 | GGTTG**c**gattcgtgaaatgtatccaaaagcgcactttgt**C**ACCAT |
|  |  |  | 363-364 | delG | 1 | CGCTGGTTGA > CGCTGTTGA |
|  |  |  | 394-401 | del | 1 | CGCAA**gat**acctg**GAT**TGAAC |
|  |  |  | 406 | G>A | 1 | GATTGAACA > GATTAAACA |
|  |  |  | 406 | G>T | 1 | GATTGAACA > GATTTAACA |
|  |  |  | 410 | A>G | 1 | GAACAGCCG > GAACGGCCG |
|  |  |  | 416-417 | GG>AA | 1 | CCGTGGGATA > CCGTAAGATA |
|  |  | S29982-2 | 8 | A>G | 1 | AGCGAAAAA > AGCGGAAAA |
|  |  |  | 27 | G>A | 1 | CCTGGGACA > CCTGAGACA |
|  |  |  | 34-35 | delT | 2 | CATGTTGCAG > CATGTGCAG |
|  |  |  | 86-87 | GG>AA | 1 | CAATGGAAAG > CAATAAAAAG |
|  |  |  | 115 | G>T | 1 | TGGCGGTCT > TGGCTGTCT |
|  |  |  | 124-125 | delC | 1 | GGTACCGGGT > GGTACGGGT |
|  |  |  | 127 | G>C | 1 | ACCGGGTGC > ACCGCGTGC |
|  |  |  | 187 | T>G | 1 | CAGCTACGA > CAGCGACGA |
|  |  |  | 189-192 | del | 2 | AGCTA**c**gat**C**ACGAC |
|  |  |  | 406 | delG | 1 | GATTGAACA > GATTAACA |
|  |  |  | 406 | G>T | 1 | GATTGAACA > GATTTAACA |
|  |  |  | 414 | delG | 1 | AGCCGTGGG > AGCCTGGG |
|  |  |  | 417 | G>A | 1 | CGTGGGATA > CGTGAGATA |
|  |  |  | 418 | G>T | 1 | GTGGGATAT > GTGGTATAT |
|  |  |  | complex 16 | | 1 | 19 G > C; 437 delT |
|  |  |  | complex 17 | | 1 | 27 G > A; 363 delG; 396 T > A |
|  |  |  | complex 18 | | 1 | 170-177 CCGTTTGT > GGG |
|  |  | S29982-3 | 86 | G>A | 1 | CAATGGAAA > CAATAGAAA |
|  |  |  | 202 | C>T | 1 | CAACCAGCG > CAACTAGCG |
|  |  |  | 274 | G>A | 1 | GGTGGATAC > GGTGAATAC |
|  |  |  | 402 | G>T | 2 | CCTGGATTG > CCTGTATTG |
|  |  |  | 415 | T>C | 1 | GCCGTGGGA > GCCGCGGGA |
|  | 65 kcal | S29981-1 | 143 | G>A | 1 | GCGCGTGAA > GCGCATGAA |
|  |  |  | 184 | A>G | 1 | TTCCAGCTA > TTCCGGCTA |
|  |  |  | 237 | delC | 1 | AAGGCGATG > AAGGGATG |
|  |  |  | 269 | T>C | 1 | GACCTGGTG>GACCCGGTG |
|  |  | S29981-2 | 8-12 | delA | 1 | AGCGAAAAATACA > AGCGAAAATACA |
|  |  |  | 25 | T>C | 1 | CACCTGGGA > CACCCGGGA |
|  |  |  | 64 | C>T | 1 | AAGCCGACT > AAGCTGACT |
|  |  |  | 110 | G>A | 1 | AGCCGTGGC > AGCCATGGC |
|  |  |  | 182-183 | CC>AA | 2 | ATTTCCAGC > ATTTAAAGC |
|  |  |  | 315-318 | insA | 1 | TATCCAAAAGCGCA>TATCCAAAAAGCGCA |
|  |  |  | 343 | A>G | 1 | CGCAAAACC > CGCAGAACC |
|  |  |  | 417 | G>C | 1 | CGTGGGATA > CGTGCGATA |
|  |  |  | complex 19 | | 1 | 7 G > T; 52 A > G |
|  |  |  | complex 20 | | 1 | 7 G > T; 130 G > C |
|  |  | S29981-3 | 95-96 | delT | 1 | GGCATTATTG > GGCATATTG |
|  |  |  | 110 | G>A | 1 | AGCCGTGGC > AGCCATGGC |
|  |  |  | 115 | G>A | 2 | TGGCGGTCT > TGGCAGTCT |
|  |  |  | 186 | C>A | 1 | CCAGCTACG > CCAGATACG |
|  |  |  | 315-318 | delA | 1 | ATCCAAAAGCGC > ATCCAAAGCGC |
|  |  |  | 391 | C>T | 1 | CCCGCAAGA > CCCGTAAGA |
|  |  |  | 406 | G>T | 1 | GATTGAACA > GATTTAACA |
|  |  |  | 409 | C>T | 1 | TGAACAGCC > TGAATAGCC |
|  |  |  | 417-418 | insA | 1 | CGTGG\|GATAT > CGTGGAGATAT |
|  |  |  | complex 21 | | 1 | 358-368 CCGCTGGTTGA > TTTT |
| 100 d | 95 kcal | S32836 | 125 | C>G | 2 | GTACCGGGT > GTACGGGGT |
|  |  |  | 244 | G>T | 1 | TGGCGAAGG > TGGCTAAGG |
|  |  |  | 406 | G>A | 1 | GATTGAACA > GATTAAACA |
|  |  |  | complex 22 | | 1 | 275 A > G; 415-416 insG |
|  |  | S32837 | 115 | G>A | 1 | TGGCGGTCT > TGGCAGTCT |
|  |  |  | 418 | G>T | 1 | GTGGGATAT > GTGGTATAT |
|  |  | S32838 | 8-12 | delA | 1 | AGCGAAAAATACA > AGCGAAAATACA |
|  |  |  | 86 | G>A | 1 | CAATGGAAA > CAATAGAAA |
|  |  |  | 342-345 | delA | 1 | TTCGCAAAACCGGC>TTCGCAAACCGGC |
|  |  |  | complex 23 | | 1 | 33-38 del(GTTGCA); 115 delG |
|  |  | S32839 | 329 | T>A | 1 | TTTGTCACC > TTTGACACC |
|  |  |  | 419 | A>T | 1 | TGGGATATG > TGGGTTATG |
|  |  |  | complex 24 | | 1 | 125 C > G; 416 delG |
|  |  | S32840 | 25 | T>G | 1 | CACCTGGGA > CACCGGGGA |
|  |  |  | 116 | G>A | 1 | GGCGGTCTG > GGCGATCTG |
|  |  |  | 406 | G>T | 1 | GATTGAACA > GATTTAACA |
|  | 65 kcal | S32841 | 137 | T>C | 4 | TTACTGGCG > TTACCGGCG |
|  |  |  | 176 | G>T | 1 | GTTTGTATT > GTTTTTATT |
|  |  |  | 406 | G>T | 1 | GATTGAACA > GATTTAACA |
|  |  |  | complex 25 | | 1 | 110 G > A; 137 T > C; 362-363 insG; 415-416 insG; 422-423 insG; 436 G > A |
|  |  |  | complex 26 | | 1 | 245-261 AAGGCTTCATCGTTATT > G |
|  |  |  | complex 27 | | 1 | 413 C > A; 451-416 insG |
|  |  | S32842 | 115 | G>A | 1 | TGGCGGTCT > TGGCAGTCT |
|  |  |  | 179 | T>A | 1 | TGTATTTCC > TGTAATTCC |
|  |  |  | 202 | C>T | 1 | CAACCAGCG > CAACTAGCG |
|  |  |  | 375 | T>A | 1 | ACTATGTTG > ACTAAGTTG |
|  |  |  | 415 | T>A | 1 | GCCGTGGGA > GCCGAGGGA |
|  |  | S32843 | 82 | C>T | 1 | TGAACAATG > TGAATAATG |
|  |  |  | 277 | A>C | 1 | GGATACCGG > GGATCCCGG |
|  |  |  | 386 | delT | 1 | GATATCCCG > GATACCCG |
|  |  | S32344 | 64 | C>T | 1 | AAGCCGACT > AAGCTGACT |
|  |  |  | 401 | G>A | 1 | ACCTGGATT > ACCTAGATT |
|  |  |  | complex 28 | | 1 | 19 G > C; 237 delC |
|  |  | S32845 | 107 | G>A | 1 | GTAAGCCGT > GTAAACCGT |
|  |  |  | 110 | G>A | 2 | AGCCGTGGC > AGCCATGGC |
|  |  |  | 110 | G>T | 1 | AGCCGTGGC > AGCCTTGGC |
|  |  |  | 115 | G>A | 1 | TGGCGGTCT > TGGCAGTCT |
|  |  |  | 161 | delA | 2 | CGTCATGTC > CGTCTGTC |
|  |  |  | 173-197 | del | 1 | TACCGtttgtatttccagctacgatcacgaCAACC |
|  |  |  | 176 | G>T | 1 | GTTTGTATT > GTTTTTATT |

^a, b^ See Supplementary Table 1.
